# Survival Contradiction in Stage II, IIIA, And IIIB Colon Cancer: A Surveillance, Epidemiology, and End Result-Based Analysis

**DOI:** 10.1155/2022/4088117

**Published:** 2022-11-16

**Authors:** Yijun Li, Rui Hua, Jianjun He, Huimin Zhang

**Affiliations:** ^1^Department of Breast Surgery, The First Affiliated Hospital of Xi'an Jiaotong University, Xi'an, China; ^2^Department of Cardiovascular Medicine, The First Affiliated Hospital of Xi'an Jiaotong University, Xi'an, China

## Abstract

There exists an inconsistency between stage and survival in the current American Joint Committee on Cancer (AJCC) staging system for colon cancer. In this study, we compared the clinicopathological characteristics and prognosis of colon cancer patients with stage II, IIIA, and IIIB disease based on the surveillance, epidemiology, and end results (SEER) database. Kaplan-Meier analysis was used to generate overall survival (OS) and cancer-specific survival (CSS) curves. The Cox regression was employed to identify risk factors. The competing risk model was completed by the cumulative incidence function and Gray's test. In the final population of 31,361 colon cancer patients, Kaplan-Meier curve analysis showed that stage IIIA had the highest OS and CSS, followed by stage IIA and IIIB, and IIB and IIC showed the worst OS and CSS. In the Cox model, the stage was proven to be an independent prognostic factor. In the competing risk model, stage IIIA colon cancer patients had the lowest 5-year cancer-specific death rate in stages II, IIIA, and IIIB. In conclusion, the prognosis of colon cancer patients in stage IIA was worse than that of patients in stage IIIA, while the survival rate of stage IIB and IIC was lower than that of stage IIIB.

## 1. Introduction

Colorectal cancer (CRC) is the second leading cause of cancer-related death in the United States. It is estimated that in 2022, approximately 151,030 people will be diagnosed with CRC, and 52,580 people will die from this disease [1]. The tumor, node, and metastasis (TNM) stage of the American Joint Committee on Cancer (AJCC) is the most widely used CRC staging system. In the 6th edition of the AJCC staging standard, stage II was divided into IIA and IIB for the first time, and stage III included IIIA, IIIB, and IIIC. The concept of stage IIC was added, and the subgroups of stage III were adjusted in the subsequent 7th edition [2]. The provisions for stages II and III in the latest 8th edition have not been changed compared with those in the 7th edition. With the deepening understanding of CRC, the specific substage has been redivided, but the criterion for distinguishing stage II from III has always been the presence of lymph node metastasis.

There exist controversies about the current staging system for CRC, and some researchers have reported inconsistencies between stage and survival. For example, in some studies, the prognosis of stage IIIA rectal cancer patients was better than that of stage II, and they believed this phenomenon was related to the deficiency of the number of lymph nodes harvested (LNH) [3, 4]. In another study comparing 359 colon cancer patients, Kim et al. found that the oncological outcome of T4N0 patients was worse than that of T1-2N1 [5]. Because of the standardized application of total mesorectal excision (TME) and complete mesocolic excision (CME), the treatment of lymph nodes in stage III colon cancer patients was satisfactory, and the prognosis was improved [6]. However, stage II patients have a higher T stage than stage III, which means a higher tumor load. Patients with locally advanced (especially T4) tumors have higher rates of local recurrence and peritoneal metastasis. It has been reported that peritoneal metastasis occurs in 15–20% of T4 colon cancer operations and seriously affects prognosis [7].

Due to the lack of multicenter, large population retrospective studies, the difference in the prognosis of patients with stage II, IIIA, and IIIB colon cancer is still unclear. The surveillance, epidemiology, and end results (SEER) database can provide a wealth of information about the pathology and survival of cancer patients. Therefore, in this research, we used the SEER database to compare the clinicopathological characteristics and prognosis of colon cancer patients with stage II, IIIA, and IIIB in the real world.

## 2. Materials and Methods

### 2.1. Data Resource

The SEER program, established by the National Cancer Institute, collects demographic, clinicopathological, and survival information on tumors in representative geographic regions of the United States. The SEER database has gathered and published information about cancer incidence and survival covering approximately 34.6% of the population in the United States [8] and is the largest public cancer database in the world. We extracted the relevant data using SEER*∗*Stat 8.3.8 software (https://seer.cancer.gov/seerstat/). The Ethics Committee of the First Affiliated Hospital of Xian Jiaotong University exempted the review of the study because the SEER database is publicly available.

### 2.2. Population Selection

**Figure 1:**
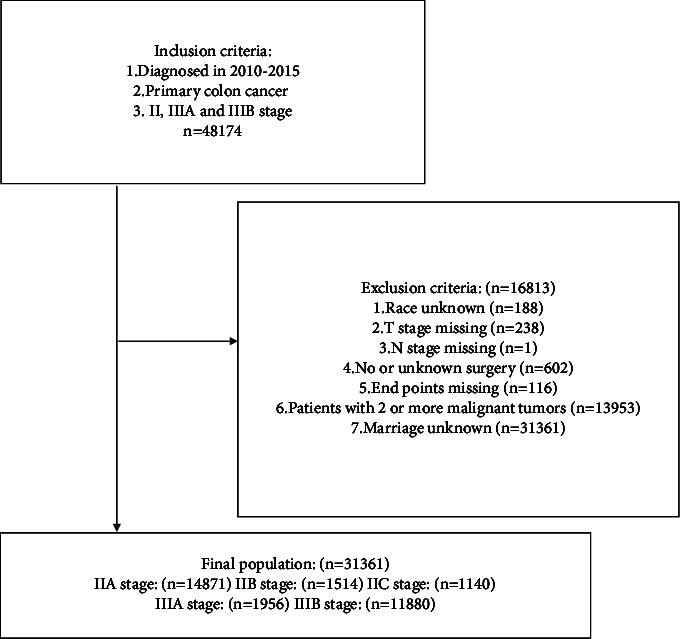
The selection criteria of the study population from the surveillance, epidemiology and end results database.

According to the SEER database, we accepted the 8th edition AJCC staging system to determine the tumor stage and identified 48,174 patients with stage II, IIIA, or IIIB primary colon cancer between 2010 and 2015. The exclusion criteria were as follows: (1) race unknown; (2) T stage missing; (3) N stage missing; (4) no or unknown surgery; (5) endpoints missing; (6) patients with 2 or more malignant tumors; and (7) marriage unknown. After the final screening, a total of 31,361 patients met the criteria. There were 14,871 patients in stage IIA, 1,514 patients in stage IIB, 1,140 patients in stage IIC, 1,956 patients in stage IIIA, and 11,880 patients in stage IIIB. The detailed selection process is shown in Figure 1.

### 2.3. Variables and Outcome

The following clinicopathological features were collected according to the SEER database: sex, race, age, histological type, primary site, LNH, TNM stage, radiation state, chemotherapy state, survival months, vital status, cause of death, and marital status. On the basis of the 8th AJCC staging system, T3N0M0 is defined as stage IIA, T4aN0M0 is defined as stage IIB, T4bN0M0 is defined as stage IIC, T1-2N1M0 or T1N2aM0 is defined as stage IIIA, and T3-4aN1M0, T2-3N2aM0, or T1-2N2bM0 is defined as stage IIIB. The ascending colon, hepatic flexure of the colon, and transverse colon are considered to be the right colon. Spiral flexibility of the colon, descending colon, and sigmoid colon are regarded as the left colon. Overall survival (OS) was measured from the date of diagnosis to the date of death. Cancer-specific survival (CSS) and cancer-specific death (CSD) referred to the date of diagnosis to the date of death from cancer.

### 2.4. Statistical Analysis

The baseline characteristics of the patients were summarized by the frequency table, and the differences were analyzed by the chi-square test. Kaplan-Meier curves were used to analyze and generate OS and CSS curves, while the log-rank test was performed to detect the significant differences among the groups. The Cox proportional hazard model was employed to identify the risk factors affecting prognosis. The competing risk model analysis was completed by the cumulative incidence function (CIF) and Gray's test. CIF is the probability of competitive events, and Gray's test is aimed at estimating the difference between groups. In all statistical tests, bilateral *P* < 0.05 was considered statistically significant. SPSS (version 22.0, IBM Corporation, Armonk, NY, USA) and R (version 3.6, The R Foundation for Statistical Computing, Vienna, Austria) were applied to complete the calculation.

## 3. Results

### 3.1. Patient Characteristics

Of the 31,361 patients recruited with a 44-month median follow-up time (interquartile range, 26–64 months), 47.4% were in stage IIA, 4.8% were in stage IIB, and 3.6% were in stage IIC. Stage IIIA accounted for 6.2%, and stage IIIB accounted for 37.9%. In total, 15,674 (50.0%) patients were female, and 15,687 (50.0%) patients were male. Among all patients, 24,275 (77.4%) were white, and 17,131 (54.6%) were married. Regarding the histological type, 28,168 (89.8%) patients had adenocarcinoma. A total of 15,715 (50.1%) had tumors located in the right colon, while 15,646 (49.9%) had tumors located in the left colon. The number of LNH more than 12 was more common (87.3%). A total of 442 (1.4%) patients were treated with radiotherapy, and 11,697 (37.3%) were treated with chemotherapy. Among the 5 groups, significant differences (*P* < 0.05) were found in age, race, histological type, initial diagnosed site, radiotherapy and chemotherapy, and marital status (Table 1).

### 3.2. Kaplan-Meier Curve Analysis

**Figure 2:**
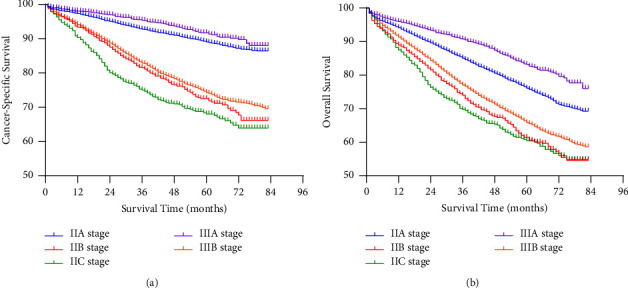
Kaplan-meier curve analyses of CSS and OS in colon cancer patients. CSS: cancer-specific survival, OS: overall survival.

**Figure 3:**
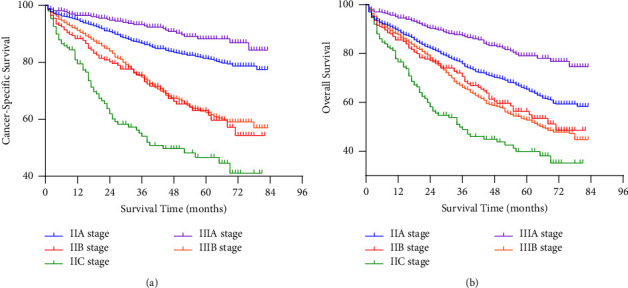
The CSS and OS of colon cancer patients with fewer than 12 lymph nodes harvested. CSS: cancer-specific survival, OS: overall survival.

**Figure 4:**
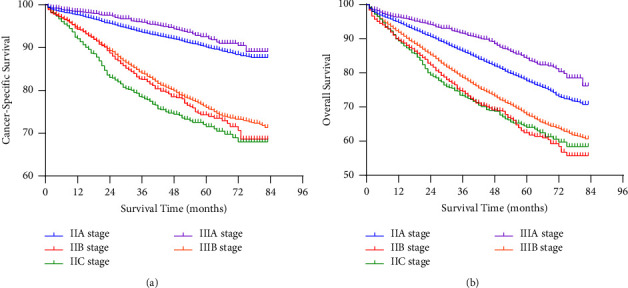
The CSS and OS of colon cancer patients with at least 12 lymph nodes harvested. CSS: cancer-specific survival, OS: overall survival.

In the final population of 31,361, 7,478 died during follow-up, of which 4,498 died of colon cancer and 2,980 died of other causes. The Kaplan-Meier curve was drawn to display the survival rate of colon cancer in stages IIA, IIB, IIC, IIIA, and IIIB (Figure 2). Figure 2(a) shows the CSS of patients enrolled in the study. The 5-year CSS rate of stage IIIA was 91.0%, followed by stages IIA and IIIB (CSS was 87.6% and 72.9%, respectively). The patients with stage IIB and IIC disease had worse CSS than the other subgroups, with 5-year CSS rates of 68.9% and 65.6% (*P* < 0.001). Figure 2(b) shows the OS rate of patients with different stages. Similar to CSS, IIB and IIC stage had the worst survival, with 5-year OS rates of 58.2% and 57.5%, respectively. The 5-year OS rates of IIA and IIIB were 74.2% and 64.0%, respectively. Stage IIIA had the best CSS, for which the 5-year CSS rate was 82.7% (*P* < 0.001). The AJCC suggests that patients with colon cancer should evaluate at least 12 lymph nodes to determine the stage. Therefore, we divided the whole population into LNH ≥ 12 and LNH < 12 subgroups to explore the relationship between stage and survival under the condition of the different number of lymph nodes examined. According to the Kaplan-Meier survival analysis, in the LNH < 12 subgroup, either for CSS (Figure 3(a)) or OS (Figure 3(b)), stage IIA tended to have worse survival rates than IIIA, while the prognosis of stage IIIB was better than that of stage IIB and IIC. The same tendency was also shown in the LNH ≥ 12 subgroup (Figure 4). Among patients with stages II, IIIA, and IIIB disease, those with stage IIIA disease had the best prognosis, while those with stage IIB and IIC disease had the lowest survival rates.

### 3.3. Cox Proportional Hazard Model

To further explore the influence of multiple factors on CSS, we used the Cox proportional risk model to evaluate risk factors and protective factors (Table 2). Univariate Cox model analysis showed that female sex, older age, black race, LNH < 12, no chemotherapy, and unmarried status were significant risk factors for survival (hazard ratio [HR] > 1, *P* < 0.05). In contrast, men, younger age, white and other races, LNH ≥ 12, chemotherapy, and married status tended to have higher survival rates (hazard ratio [HR] < 1, *P* < 0.05). Regarding the influence of stage on CSS, compared with IIIA, patients in stage IIA tended to have worse CSS (HR: 1.550, 95% CI: 1.290–1.862, *P* < 0.001). The HR of patients in stage IIIB was 3.480 (95% CI: 2.905–4.169, *P* < 0.001), while the prognosis of stage IIB and IIC was the worst, with HR values of 4.232 (95% CI: 3.444–5.200, *P* < 0.001) and 5.163 (95% CI: 4.192–6.360, *P* < 0.001), respectively. Multivariate Cox analysis was then used to identify independent risk factors for CSS in colon cancer. TNM stage was still proven to be an independent prognostic factor for CSS after adjusting for sex, age, race, histological type, initial diagnosed site, LNH, radiotherapy, chemotherapy, and marital status. Compared to patients with stage IIIA, there was no significant difference in CSS for stage IIA colon cancer (HR: 1.028, 95% CI: 0.854–1.239, *P*=0.769). The CSS of stage IIB and IIC was worse than that of IIIA, with HRs of 3.166 (95% CI: 2.573–3.895, *P* < 0.001) and 4.314 (95% CI: 3.499–5.318, *P* < 0.001), respectively. Similarly, the multivariate Cox analysis also suggested that the CSS of IIIB was relatively poor (HR: 3.492, 95% CI: 2.914–4.185, *P* < 0.001). In addition, race, age, histological type, LNH, chemotherapy, and marital status were also independently associated with CSS (*P* < 0.001).

### 3.4. Competing Risk Model and the Gray's Test

**Figure 5:**
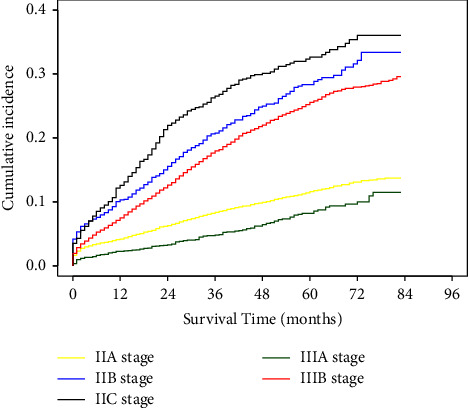
Cumulative incidence of colon cancer-specific deaths in stage II, IIIA and IIIB colon cancer patients.

Among 7,478 deaths in the whole cohort, 60.15% (4,498/7,478) were CSD, and 39.85% (2,980/7,478) were non-CSD. To reduce the bias caused by other causes of death, we constructed a competing risk analysis model investigating the effect of TNM stage on CSD. As shown in Figure 5, after excluding the effect of non-CSD on survival, stage IIIA had the lowest 5-year CSD among all stages analyzed (8.21%, Gray's test, *P* < 0.001). In stage IIA, 11.59% of patients died in 5 years. The cumulative CSD rates of stage IIB (28.34%, Gray's test, *P* < 0.001) and IIC (32.64%, Gray's test, *P* < 0.001) were both higher than that of IIIB (25.57%, Gray's test, *P* < 0.001).

## 4. Discussion

In this study, we evaluated the survival of 31,361 patients with stage II, IIIA, and IIIB colon cancer and found a contradiction between the prognosis and stage. Stage IIIA had the lowest mortality rate, and stage IIIB had better survival than IIB and IIC. The Kaplan-Meier method, log-rank test, and Cox regression are widely used in survival analysis. However, these statistical methods only involve one type of endpoint. When multiple endpoints exist, competition events and events of interest form a competition relationship, which makes Kaplan-Meier analysis and Cox regression overestimate the probability of events of interest or even obtain the opposite conclusion [9]. Therefore, a competing risk model is needed to provide a better clinical prediction [10]. After Gray's test, when the risk of death from other causes was removed, we still obtained a similar survival tendency, which further confirmed our conclusion. To our knowledge, this is the first and largest population-based study to determine the relationship between survival and stage in patients with stage II, IIIA, and IIIB colon cancer.

It has been reported that the survival rate of rectal cancer patients in stage IIA is lower than that of patients with stage IIIA when there is not enough lymph node collection, and this phenomenon may be related to stage migration [4]. Stage migration refers to when the number of lymph nodes examined is insufficient; it may be misdiagnosed as “lymph node-negative” and reduce the stage, thus lacking necessary adjuvant treatment and resulting in poor prognosis [11–13]. Therefore, in our study, the population was divided into LNH ≥ 12 and LNH < 12 subgroups. The results showed that even in the LNH < 12 subgroup, the prognosis of the IIIA stage was better than that of the II and IIIB stage, which indicated that there was no significant correlation between this survival contradiction and the number of LNH in colon cancer.

Although the AJCC staging system of CRC has been constantly modified, the over-emphasis on the N stage has always existed in various versions [14–16]. For a long time, as long as lymph node metastasis is detected, it will be cataloged into stage III, regardless of the size of the primary tumor. It has been confirmed that the T stage has a greater proportion of influence than the N stage in CRC [17]. Therefore, considering the contradiction between colon cancer stage and survival in the 8th edition, the AJCC staging system needs to reconsider the weight of the T and N stages and redefine the classification criteria of colon cancer stages II and III.

The priority of chemotherapy in stage III is considered to be a possible reason for the better prognosis of stage III compared with T4N0 colon cancer patients [18, 19]. Stage III patients are often recommended to have a higher rate of adjuvant chemotherapy, and postoperative adjuvant systemic therapy can significantly improve the prognosis [20, 21]. With the emergence of various new drugs, as well as the wide application of immunotherapy and targeted therapy [22–25], the prognosis of stage III patients is becoming increasingly ideal. In our cohort, stage IIIA had the highest chemotherapy acceptance rate (67.7%), and the percentage of patients undergoing chemotherapy in stage IIIB (62.4%) was also higher than that in stages IIB (34.3%) and IIC (46.8%). Therefore, the better prognosis of stage III may be attributed to the fact that most patients with stage III colon cancer received adjuvant chemotherapy. Whether patients with stage II colon cancer should receive standard chemotherapy deserves further discussion in the latest guidelines.

The difference in biological characteristics may be the internal mechanism of the survival contradiction [26–28]. Higher T staging not only means a larger primary tumor but also reflects stronger aggressiveness [29]. At the same time, the ratio of microsatellite instability [30–32] and perineural invasion in T4N0 colon cancer was significantly increased [33], suggesting that stage IIB and stage IIC tumors may have different biological behaviors compared with stage III.

There are some limitations in our study. First, as a retrospective study, the inevitable selection bias should not be ignored. Second, the SEER database does not include information on specific cancer-related biomarkers, which is very important for prognosis prediction. Third, we only studied the data from 2010 to 2015, which is a relatively short follow-up period, and a longer follow-up time is needed to confirm our results in the future.

## 5. Conclusions

The prognosis of colon cancer patients in stage IIA was worse than that of patients in stage IIIA, while the survival rate of colon cancer patients with stage IIB and IIC was lower than that of patients in stage IIIB, and this contradiction between survival and stage was not related to insufficient LNH. In the future, we may need to evaluate and modify the AJCC stage of colon cancer according to the prognostic information to determine a more accurate and suitable new staging system. This is a retrospective study, and large prospective studies are needed to verify our results.

## Figures and Tables

**Table 1:** Patient characteristics with stage II, IIIA and IIIB colon cancer in the SEER database.

Characteristics	Total [*N* (%)]	IIA	IIB	IIC	IIIA	IIIB	*P*
*N*	31361	14871 (47.4)	1514 (4.8)	1140 (3.6)	1956 (6.2)	11880 (37.9)	
Sex							0.082
Female	15674 (50.0)	7359 (49.5)	762 (50.3)	603 (52.9)	950 (48.6)	6000 (50.5)	
Male	15687 (50.0)	7512 (50.5)	752 (49.7)	537 (47.1)	1006 (51.4)	5880 (49.5)	
Age at diagnosis (yr)							<0.001
≤60	9709 (31.0)	4024 (27.1)	434 (29.7)	374 (32.8)	795 (40.6)	4082 (34.4)	
>60	21652 (69.0)	10847 (72.9)	1080 (71.3)	766 (67.2)	1161 (59.4)	7798 (65.6)	
Race							<0.001
White	24275 (77.4)	11800 (79.3)	1200 (79.3)	913 (80.1)	1434 (73.3)	8928 (75.2)	
Black	3823 (12.2)	1680 (11.3)	150 (9.9)	139 (12.2)	269 (13.8)	1585 (13.3)	
Other	3263 (10.4)	1391 (9.4)	164 (10.8)	88 (7.7)	253 (12.9)	1367 (11.5)	
Histological type							<0.001
Adenocarcinoma	28168 (89.8)	13333 (89.7)	1302 (86.0)	963 (84.5)	1851 (94.6)	10719 (90.2)	
Other	3193 (10.2)	1538 (10.3)	212 (14.0)	177 (15.5)	105 (5.4)	1161 (9.8)	
Initial diagnosed site							<0.001
Right colon	15715 (50.1)	8321 (56.0)	730 (48.2)	436 (38.2)	699 (35.7)	5529 (46.5)	
Left colon	15646 (49.9)	6550 (44.0)	784 (51.8)	704 (61.8)	1257 (64.3)	6351 (53.5)	
LNH							<0.001
<12	3969 (12.7)	1767 (11.9)	229 (15.1)	171 (15.0)	375 (19.2)	1427 (12.0)	
≥12	27392 (87.3)	13104 (88.1)	1285 (84.9)	969 (85.0)	1571 (80.8)	10453 (88.0)	
Radiotherapy							<0.001
Yes	442 (1.4)	96 (0.6)	47 (3.1)	121 (10.6)	18 (0.9)	160 (1.4)	
No/unknown	30919 (98.6)	14775 (99.4)	1467 (96.9)	1019 (89.4)	1938 (99.1)	30919 (98.6)	
Chemotherapy							<0.001
Yes	11697 (37.3)	1902 (12.8)	519 (34.3)	533 (46.8)	1324 (67.7)	7419 (62.4)	
No/unknown	19664 (62.7)	12969 (87.2)	995 (65.7)	607 (53.2)	632 (32.3)	4461 (37.6)	
Marriage status							<0.001
Married	17131 (54.6)	8030 (54.0)	781 (51.6)	530 (46.5)	1223 (62.5)	6567 (55.3)	
Unmarried/DSW	14230 (45.4)	6841 (46.0)	733 (48.4)	610 (53.5)	733 (37.5)	5313 (44.7)	

Note. LNH: number of lymph nodes harvested; DSW: divorced & separated &widowed.

**Table 2:** Cox regression model analyses of cancer-specific survival in stage II, IIIA and IIIB colon cancer.

Variable	Univariable analysis			Multivariable analysis		
	HR	95% CI	*P*	HR	95% CI	*P*
TNM stage			<0.001			<0.001
IIIA	1			1		
IIA	1.550	1.290–1.862	<0.001	1.028	0.854–1.239	0.769
IIB	4.232	3.444–5.200	<0.001	3.166	2.573–3.895	<0.001
IIC	5.163	4.192–6.360	<0.001	4.314	3.499–5.318	<0.001
IIIB	3.480	2.905–4.169	<0.001	3.492	2.914–4.185	<0.001
Sex			0.002			
Female	1					
Male	0.913	0.861–0.968				
Age at diagnosis (yr)			<0.001			<0.001
≤60	1			1		
>60	2.140	1.986–2.306		1.830	1.695–1.977	
Race			<0.001			<0.001
White	1			1		
Black	1.110	1.019–1.210	0.017	1.094	1.003–1.193	0.042
Other	0.796	0.715–0.885	<0.001	0.786	0.706–0.874	<0.001
Histological type			<0.001			<0.001
Adenocarcinoma	1			1		
Other	1.323	1.211–1.444		1.230	1.126–1.343	
Initial diagnosed site		0.020				
Right colon	1					
Left colon	0.933	0.880–0.989				
LNH			<0.001			<0.001
<12	1			1		
≥12	0.529	0.492–0.568		0.543	0.505–0.584	
Radiotherapy			0.321			
Yes	1					
No/unknown	0.893	0.714–1.117				
Chemotherapy			<0.001			<0.001
Yes	1			1		
No/unknown	1.538	1.444–1.639		2.139	1.992–2.296	
Marriage status			<0.001			<0.001
Married	1			1		
Unmarried/DSW	1.684	1.587–1.786		1.494	1.408–1.587	

Note: LNH: number of lymph nodes harvested; DSW: divorced & separated &widowed.

## Data Availability

The datasets generated during and/or analyzed during the current study are available in the SEER repository [https://seer.cancer.gov/seerstat/].
